# SRDFNet: Semantic Refinement and Differential Features for High-Resolution Change Detection

**DOI:** 10.3390/s26113427

**Published:** 2026-05-28

**Authors:** Wenbo Zhao, Donghua Lu, Yingjun Zhao, Keyue Chen

**Affiliations:** Beijing Research Institute of Uranium Geology, Beijing 100029, China; zhaowenbo@briug.cn (W.Z.); zhaoyingjun@briug.cn (Y.Z.); chenkeyue@briug.cn (K.C.)

**Keywords:** remote sensing, deep learning, semantic change detection, feature refinement, multi-task learning

## Abstract

To address misclassification and reduced accuracy in semantic change detection caused by class imbalance and variable object sizes, this paper improves BGSNet and proposes a new change detection network, SRDFNet (Semantic Refinement and Differential Features). Based on BGSNet’s framework, it introduces three complementary modules: (1) a hierarchical graph module (HGM) that converts multi-scale feature maps into compact semantic graph nodes, using graph attention for intra-layer and cross-level semantic interaction to enhance topological relationship perception; the HGM mitigates the effects of class imbalance by compacting multi-scale features into semantic nodes; (2) a difference enhancement (DE) module that extracts multi-receptive-field difference information from bi-temporal concatenated features via multi-scale parallel convolution branches; (3) a semantic refine (SR) module that performs lightweight residual refinement on bi-temporal semantic features to improve the segmentation accuracy. The DE and SR modules mitigate the degradation in semantic segmentation accuracy caused by variable object sizes. It is trained and tested with BGSNet and three other models on the SECOND and HRSCD datasets. For the SECOND dataset, in terms of five quantitative indicators, namely OA, mIoU, SeK, F1 and recall, SRDFNet achieves 87.64%, 70.31%, 20.36%, 60.25% and 65.27%, respectively. Compared with BGSNet, it gains performance increases of 1.34%, 0.73%, 1.44%, 0.81% and 2.72%, respectively. For the HRSCD dataset, SRDFNet achieves 98.13% (OA), 52.67% (mIoU), 73.77% (SeK), 88.86% (F1) and 88.18% (recall), ranking first among the four methods. Compared with BGSNet, it gains performance increases of 3.96%, 3.93%, 9.69%, 2.33% and 4.00%, respectively.

## 1. Introduction

In the field of remote sensing, change detection research refers to the process of obtaining surface change information by analyzing two images of the same area acquired at different times [1]. In applications such as land use, resource exploration and disaster monitoring, the use of remote sensing technology for the dynamic monitoring of surface cover is an extremely important technical means [2]. The class imbalance of changed objects means that a small number of categories of changed objects account for most of the samples [3], while other categories have very few samples, which will lead the model to be biased towards learning the features of the dominant categories during training, resulting in underfitting for minority categories and serious misclassification. Meanwhile, variable object sizes will make the model unable to capture consistent semantic and geometric features of objects of different scales [4] (small-sized changed objects are easily ignored due to insufficient feature extraction, while large-sized changed objects are prone to incomplete detection or false detection due to imbalance feature responses). In recent years, with the rapid development of technologies such as artificial intelligence, neural networks and large language models, deep learning has also been widely applied to task scenarios such as target recognition and change detection in the remote sensing field [3,5,6]. Due to its strong learning ability and deep mining of complex features, compared with other change detection methods, deep learning can more accurately capture change information in remote sensing images, improve the accuracy of interpretation and reduce the time of feature extraction [4,7,8].

The Transformer model is an important innovation in the field of deep learning; it mainly realizes the parallelization of sequence processing through its unique self-attention mechanism [9,10]. A classic method of applying Transformer to change detection is ChangeFormer, proposed by Bandara et al. [11], which uses a hierarchical Transformer encoder and a lightweight MLP decoder to process bi-temporal images in a Siamese architecture. Although it reduces the computational overhead, its fixed-window attention mechanism restricts cross-window feature interaction, resulting in limited capabilities for detecting irregular changes. Zhang et al. [12] combined Swin Transformer with UNet to propose SwinSUNet; it breaks free from the locality constraint of convolution but suffers from problems such as an excessively large number of parameters and insufficient utilization of shallow detailed features. Teng et al. [13] proposed SFCD, which uses Swin Transformer instead of a traditional CNN as the encoder in the feature extraction stage, exerting the advantages of Swin Transformer in small-target and local-area change detection. However, this method relies on ImageNet pre-trained weights, leading to limited generalization performance on small-sample remote sensing datasets. Guo et al. [14] proposed an iterative difference enhancement method (IDET), which enhances differential features in an iterative manner to improve the change detection accuracy, but multi-scale iterative refinement introduces extra computational overhead, and the inference efficiency needs to be improved. Yang et al. [15] proposed a Siamese encoder–decoder network based on graph context attention (GCA-SEDN). It fuses graph context attention to capture the spatial topological relationships of ground objects and eliminates the annotation dependence, making it suitable for unlabeled scenarios. However, it is designed specifically for polarimetric SAR data and has poor adaptability to optical remote sensing images. In recent years, multi-task learning and multi-scale fusion have become research hotspots in the field of semantic change detection (SCD). Traditional CNN models and their extended networks, such as LeNet-5 [16], AlexNet [17], VGG [18], ResNet [19] and DenseNet [20], which are dedicated to binary change detection tasks, have gradually failed to meet the requirements. Chen et al. [21] combined the Siamese network with the UNet model and introduced Atrous Spatial Pyramid Pooling (ASPP) to enhance the multi-scale feature detection capabilities. Pang et al. [22] proposed SCA-CDNet, a robust Siamese correlation and attention change detection network; however, this method still relies on a CNN as its main backbone, resulting in insufficient global modeling capabilities. Cui et al. [23] proposed MTSCD-Net, which adopts a Swin Transformer-based Siamese semantic perception encoder to extract bi-temporal multi-scale features, but it suffers from insufficient task interaction, weak semantic consistency constraints and the inadequate suppression of pseudo-changes and seasonal disturbances. Wang et al. [24] proposed a cross-difference semantic consistency network, which improves SCD performance by enhancing the collaboration between binary change detection and semantic segmentation subtasks and using modeled difference features to resolve the limitation of consistency in the bi-temporal feature space, but it is difficult to simultaneously balance global semantics and local details. Some studies focus on improving SCD performance through semantic enhancement and change consistency strategies. For example, methods based on SAM2 are used to extract global features to address the problems of insufficient semantic extraction and inconsistent change features [25]. To better capture surface cover features in complex scenarios, Liu et al. [26] proposed an SCD model based on spatiotemporal attention perception and multi-scale fusion to solve the problems of spatial detail loss and insufficient global feature modeling capabilities. Although such methods have achieved good results, how to effectively utilize the correlation between tasks and promote the overall performance of the model remains a challenge.

To address the above dilemma of insufficient multi-task collaboration and achieve the high-precision semantic change detection of bi-temporal remote sensing images, it is necessary to address (1) structured semantic representation in multi-scale feature fusion; (2) expressive multi-scale difference modeling that bridges the change detection and semantic segmentation branches; and (3) noise suppression in temporal cross-attention to safeguard the per-temporal semantic accuracy. We propose three collaborative improvement modules for different subtasks based on BGSNet and construct a semantic change detection network, SRDFNet (Semantic Refinement and Differential Features), oriented to multi-task joint optimization. Our research contributions are summarized as follows:

(1) We design a hierarchical graph module (HGM) to enhance the semantic structured representation in the multi-scale feature fusion stage. This improvement provides more discriminative shared feature representations for both downstream semantic segmentation and change detection subtasks, improving the collaborative effect of multi-tasks from the source.
(2) We also propose a difference enhancement (DE) module to bridge the feature gap between the change detection branch and the semantic segmentation branch. Compared with the simple pixel-wise absolute difference operation, DE can capture richer multi-scale change patterns and achieve mutual improvement between change detection and semantic segmentation tasks.
(3) In addition, we introduce a semantic refine (SR) module specifically for the end-to-end optimization of the semantic segmentation subtask. It suppresses the noise interference introduced by temporal cross-attention in the process of enhancing change information and improves the accuracy of semantic segmentation for each temporal phase.

## 2. Methods

### 2.1. BGSNet

**Figure 1:**
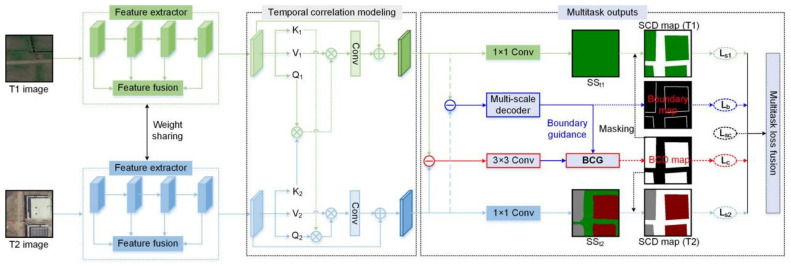
The structure of BGSNet [27].

On the basis of its existing three branches (i.e., bi-temporal semantic change detection and binary change detection tasks), the BGSNet model adds a new boundary detection branch [27,28,29] and establishes the association between boundary and change features through boundary-contextual guidance (BCG), which is the key design feature that distinguishes BGSNet from all comparable methods. As shown in Figure 1, the overall architecture of BGSNet adopts a design paradigm of a shared encoder and multi-task decoder, and it enhances the accuracy and geometric consistency of semantic change detection by jointly optimizing three interrelated tasks. The reason that BCG can improve model performance is mainly reflected in the following three aspects. (1) As an inherent geometric attribute of ground objects, the boundary has more stable semantic expression than the interior of the region and is less affected by temporal spectral changes and seasonal differences. Therefore, boundary features can provide a cross-temporal robust structural prior for change detection. (2) By taking boundary detection as an independent supervised task for joint learning, the shared encoder can be forced to learn more discriminative geometry-aware features, thereby enhancing the intra-class consistency of similar change regions. (3) Boundary semantics inherently have region division capabilities, which can be used as contextual constraints to refine the spatial details of high-level semantic difference features, effectively suppressing cross-boundary error diffusion and thus improving the boundary regularity and geometric accuracy of the detection results.

In this model structure, a Siamese feature extractor with shared weights acquires robust semantic features; the temporal correlation of bi-temporal features is modeled through the “Query–Key–Value” attention mechanism to enhance the feature consistency of invariant regions and initially suppress false changes. Among them, the Query (*Q*) is derived from the semantic features of the previous phase (T1) and is used to express the “semantic content to be queried”; the Key (*K*) is derived from the semantic features of the subsequent phase (T2) and is used for similarity matching with the Query to measure the semantic correlation strength between bi-temporal pixels; the Value (*V*) is also derived from the features of the subsequent phase (T2) and carries the semantic information actually involved in weighted aggregation. The temporal attention weight matrix is generated through the dot product operation of *Q* and *K*, and then *V* is weighted and aggregated; in this way, we can explicitly model the semantic consistency of invariant regions between the two phases. The multi-scale decoder (MSD) enhances the semantic representation of boundaries at different scales through continuous convolution; the BCG module generates refined features with boundary constraints by introducing boundary guidance.

### 2.2. SRDFNet

**Figure 2:**
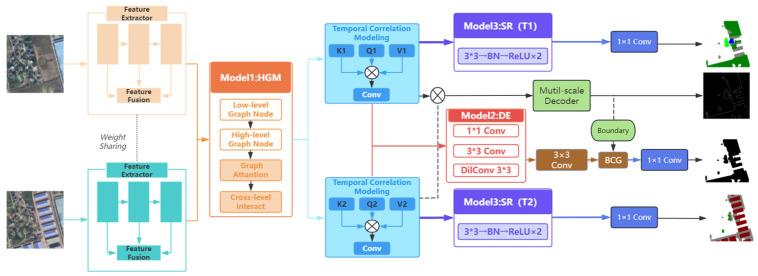
The structure of SRDFNet.

SRDFNet takes the BGSNet model as its basic framework and constructs a more targeted architecture for semantic change detection, whose overall structure is shown in Figure 2. The input bi-temporal images (T1, T2) are first fed into a weight-shared Siamese feature extractor (MFPNet-HGM) to generate multi-scale semantic features. Subsequently, the HGM module performs cross-level semantic graph interaction on the bi-temporal features. The temporal correlation modeling module then captures the temporal semantic dependencies, while the DE module simultaneously mines bi-temporal difference information through multi-scale convolutions. Finally, the SR module refines the semantic outputs, and the boundary detection branch achieves the boundary-guided fine-grained identification of changed regions via the multi-scale decoder and the BCG module, ultimately producing the bi-temporal semantic segmentation maps, the change region map and the boundary map.

SRDFNet mainly adds three core functional modules on the basis of BGSNet, namely the HGM, DE module and SR module. Among them, the HGM module focuses on addressing the core limitation that the multi-scale feature interaction process in existing models lacks in-depth semantic-level modeling. Through the construction of semantic graph nodes, the introduction of a graph attention mechanism and the design of cross-hierarchy feature interaction, it realizes the enhancement of semantic correlation and effective fusion of multi-scale features [30]. The DE module is mainly responsible for capturing multi-scale change information and can accurately mine feature differences at different scales [24], while the SR module focuses on solving the problem that the cross-attention mechanism is prone to introducing noise during the feature fusion process [28], which leads to a decline in semantic accuracy, and it effectively reduces noise interference through targeted semantic refinement operations. These three modules systematically enhance the feature complementarity between semantic change detection tasks from three key aspects—feature fusion, difference modeling and semantic refinement—thereby significantly improving the detection performance of the entire model.

#### 2.2.1. MFPNet-HGM

**Figure 3:**
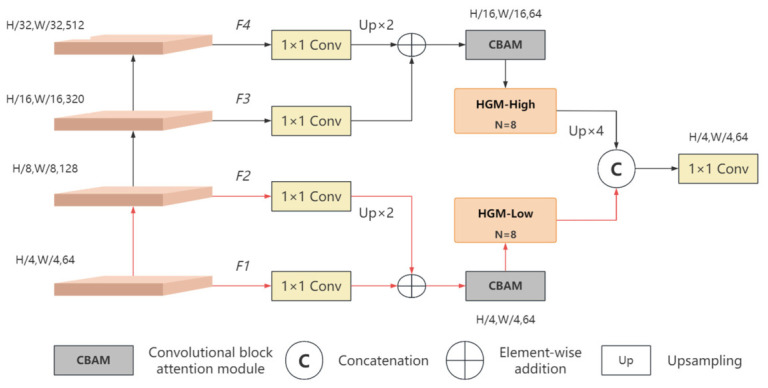
The structure of MFPNet-HGM (Red arrows indicate low-level features, while black arrows indicate high-level features).

On the basis of the original MFPNet, we introduce the HGM mechanism to construct an enhanced feature extractor, namely MFPNet-HGM, as shown in Figure 3. Specifically, we adopt a prevalent pyramid transformer (PVT-v2 [31]) to extract multi-scale features at four stages from bi-temporal remote sensing images. The obtained features are denoted as $F_{i}\in R^{C\times H\times W}(i\in 1,2,3,4)$ with spatial resolutions of 1/4, 1/8, 1/16 and 1/32 of the input size and corresponding channel dimensions of 64, 128, 320 and 512, respectively.

Low-level features *F*1 and *F*2 retain abundant spatial details and edge information, while high-level features *F*3 and *F*4 encode abstract semantic category information. The complementarity between low-level and high-level features forms the foundation of the multi-scale feature pyramid. To achieve efficient feature fusion at different semantic levels, *F*4 is first projected to a unified channel dimension (64 channels) via a 1 × 1 convolution. After double bilinear upsampling, it is added element-wise to the feature of *F*3, adjusted by a 1 × 1 convolution. The fused feature is then fed into a convolutional block attention module (CBAM [32]) for the dual recalibration of the channel and spatial dimensions, outputting high-level fused features with a resolution of *H*/16 × *W*/16 and 64 channels. Similarly, *F*1 and *F*2 are processed in a symmetric manner to obtain low-level fused features with a resolution of *H*/4 × *W*/4 and 64 channels.

To further explore category-level structured semantic relationships within fused features, we embed the hierarchical graph mutual module (HGM) after the CBAM of the two fusion branches, named HGM-Low for low-level features and HGM-High for high-level features. The node number *N* is a critical hyperparameter of the HGM, which is determined by the following three criteria: (1) matching the number of semantic categories—the node number should not be less than the category number, and appropriate redundancy is reserved to strengthen the representation capabilities; (2) matching the minimum spatial resolution—the input resolution of HGM-High is 32 × 32, so the value of *N* should be much smaller than 32; (3) computational complexity constraint—an excessively large *N* will dramatically increase the computational cost, and a balance between accuracy and efficiency should be guaranteed. Accordingly, we set the node number *N* to 8. The HGM first softly aggregates pixel-level features into *N* semantic nodes through a learnable node assignment matrix; it then performs graph attention interaction at the node level and finally re-projects the updated node features back to the spatial domain, which are added to the input features in a residual manner. The parallelly deployed HGMs at two scales capture fine-grained local correlations and coarse-grained global semantics, respectively, and realize hierarchical information coupling through cross-scale node interaction.

The output of the HGM-High module is upsampled by quatra-bilinear interpolation to restore the spatial resolution of *H*/4 × *W*/4 and concatenated with the output of the HGM-Low module in the channel dimension to generate a joint representation integrating multi-scale semantic graph structural information. The joint representation is compressed to 64 channels via a 1 × 1 convolution and finally outputs shared features with a resolution of *H*/4 × *W*/4 and 64 channels. It provides a high-quality feature representation with both spatial details and a semantic structure for the change detection branch and bi-temporal semantic segmentation branch.

#### 2.2.2. HGM

In the BGSNet model, low-level features and high-level features are fused through simple CBAM attention and concatenation, lacking explicit modeling at the semantic level. The spatial distribution relationships between different semantic categories (such as buildings, vegetation and water bodies) are not fully utilized. The HGM converts the feature map $F_{i}(i\in 1,2,3,4)$ into *N* (*N* = 8) semantic graph nodes $V\in R^{N\times C}$ and performs cross-level information interaction in the graph space:

(1) $$A_{i}\;=\;\mathrm{Softmax}\;(\mathrm{BN}\;(W_{a}F_{i}))$$

(2) $$V_{i}=\frac{A_{i}\cdot F_{i}^{T}}{\sum_{j=1}^{\mathrm{HW}}A_{j}}$$

where $W_{a}F_{i}$ indicates a 1 × 1 convolutional learnable parameter, $A_{i}$ denotes a soft assignment matrix representing the probability that each spatial position belongs to each semantic node, T denotes vector transposition, and BN indicates batch normalization. After the Softmax operation, the value range of convolutional outputs fluctuates significantly with the network depth, training stages and input samples. BN normalizes the input to a distribution with zero mean and unit variance, enabling Softmax to function within an interval with stable gradients and distinguishable feature allocation, which balances assignment sharpness and gradient flow. Then, multi-head self-attention is performed on the low-level nodes $V_{low}$ and high-level nodes $V_{high}$. In order to model the dependencies between semantic nodes within the same level, intra-graph message passing based on multi-head self-attention (MHSA) is performed on $V_{low}$ and $V_{high}$. Taking the low-level node $V_{low}$ as an example (the high-level node $V_{high}$ is processed in the same way), let *h* be the number of attention heads and *d* = *C^low^*/*h* be the dimension of each head. The formulations are defined as follows:

(3) $$Q_{low}=LN\;(V_{low}){\;W}_{Q}^{\left(low\right)},K_{low}=LN\;(V_{low})\;W_{K}^{\left(low\right)},{V^{\prime }}_{low}=LN\;(V_{low}){\;W}_{V}^{\left(low\right)}$$

(4) $$V_{low}^{attn}=Softmax(\frac{Q_{low}K_{low}^{T}}{\sqrt{d}})\;{V^{\prime }}_{low}$$

(5) $${\hat{V}}_{\mathrm{low}}=V_{low}+V_{low}^{attn}W_{O}^{\left(low\right)}$$

(6) $${\tilde{V}}_{low}={\hat{V}}_{\mathrm{low}}+FFN\;(LN\;({\hat{V}}_{\mathrm{low}}))$$

where LN indicates layer normalization; $W_{Q}^{\left(low\right)}$, $W_{K}^{\left(low\right)}$, $W_{V}^{\left(low\right)}$ and $W_{O}^{\left(low\right)}$ are learnable linear projection matrices; $\sqrt{d}$ indicates the scaling factor, which is adopted to prevent excessive dot-product values from causing Softmax gradient vanishing; FFN refers to the two-layer feed-forward network. ${\hat{V}}_{\mathrm{low}}$ and ${\tilde{V}}_{low}$ represent the intermediate and final node features after the attention residual operation and FFN residual operation, respectively.

To model the semantic consistency between high-level and low-level features, the HGM takes high-level nodes ${\tilde{V}}_{high}$ as *Q* (Query) and low-level nodes as *K* (Key) and *V* (Value) and performs one-way cross-level attention:

(7) $${\tilde{V}}_{high}^{C}={\tilde{V}}_{high}+\;Softmax\;(\frac{Q^{C}{\left(K^{C}\right)}^{T}}{\sqrt{d^{C}}}){\;V}^{C}$$

where $Q^{c}=LN({\tilde{V}}_{high})W_{Q}^{C}$, $K^{C}=LN({\tilde{V}}_{low})W_{K}^{C}$, $V^{C}=LN({\tilde{V}}_{low})W_{V}^{C}$, and $d^{C}$ denotes the scaling dimension for cross-level attention, which is set as $d^{C}$ = *C^low^*/4 in this work; ${\tilde{V}}_{high}^{C}$ represents the updated high-level nodes integrated with detailed low-level context information. Finally, the updated graph nodes are re-projected back to the spatial feature maps:

(8) $$F_{low}^{enhanced}=F_{low}+\alpha \cdot \phi \;({{(\tilde{V}}_{low})}^{T}\cdot A_{low})$$

(9) $$F_{high}^{enhanced}=F_{high}+\alpha \cdot \phi \;({\left({\tilde{V}}_{high}^{C}\right)}^{T}\cdot A_{low})$$

where ϕ indicates the projection layer, composed of a 1 × 1 convolution, BN and ReLU activation. The learnable weight α is initialized to 0, enabling this module to act as an identity mapping at the early training stage and thereby preserving the pre-trained features without destruction.

#### 2.2.3. DE Module

BGSNet uses a simple absolute difference $\left|{F_{T1}-F}_{T2}\right|$ to detect change regions. This method only captures pixel-wise amplitude differences, ignoring multi-scale contextual change information and the differences in change patterns between channels. The DE module enhances differential features through multi-scale convolution and residual fusion. First, three convolution branches with different receptive fields are used:

(10) $$F_{cat}\;=\;\mathrm{Concat}\;(F_{T1},\;F_{T2})$$

(11) $$D_{1}=\mathrm{ReLU}\;(\mathrm{BN}\;({\mathrm{Conv}}_{1\times 1}(F_{cat})))$$

(12) $$D_{3}=\mathrm{ReLU}\;(\mathrm{BN}\;({\mathrm{Conv}}_{3\times 3}(F_{cat})))$$

(13) $$D_{5}=\mathrm{ReLU}\;(\mathrm{BN}\;({\mathrm{DilConv}}_{3\times 3,\;d=2}(F_{cat})))$$

where ${\mathrm{DilConv}}_{3\times 3,\;d=2}$ indicates 3 × 3 dilated convolution with a dilation rate of 2, which is equivalent to a receptive field of 5 × 5. Combine the three branches:

(14) $$D_{fuse}\;=\;{\mathrm{Conv}}_{1\times 1}\;(\mathrm{Concat}\;(D_{1},D_{3},D_{5}))$$

where $D_{fuse}$ indicates the multi-scale difference fusion feature. Afterwards, channel attention is adopted to assign adaptive weights to each channel:

(15) $$w\;=\;\sigma \;(W_{2}\cdot \mathrm{ReLU}\;(W_{1}\cdot \mathrm{GAP}(D_{fuse})))\;\in R^{C\times 1\times 1}$$

(16) $$\hat{D\;}=w\;⊙\;D_{fuse}$$

where GAP denotes global average pooling. $W_{1}\in R^{C/4\times C}\;$ and $W_{2}\in R^{C\times C/4}$ are fully connected layers, σ represents the Sigmoid activation function, and ⊙ denotes element-wise multiplication, while $\hat{D}$ indicates the enhanced difference feature.

For residual fusion, the scaling factor β is initialized to 0, and it degrades to an absolute difference in the early stage of training to ensure the original performance of the baseline.

(17) $$D_{\mathrm{out}}=\left|{F_{T1}-F}_{T2}\right|+\beta \cdot \hat{D}$$

where $D_{\mathrm{out}}$ denotes the output difference feature. When β = 0, $D_{\mathrm{out}}$ represents the original difference feature. After residual fusion, the value of β increases automatically to accelerate the convergence of the model. Meanwhile, as the model gradually converges, the value of $\hat{D}$ declines steadily, which guarantees the stability of model training.

**Figure 4:**
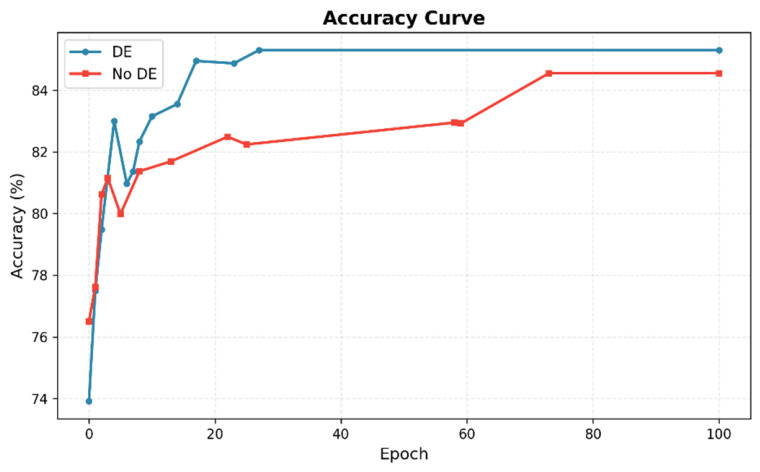
Accuracy convergence curves of DE and no-DE strategies with training epochs.

To investigate the impact of the learnable parameter β on model convergence, we conducted experiments examining the accuracy convergence curves of the model with and without the DE strategy, as illustrated in Figure 4.

The horizontal axis represents the training epoch, and the vertical axis denotes the model accuracy. It can be observed that the model accuracy under both strategies improves rapidly in the initial training stage and gradually converges with the increase in epochs. Compared with the strategy without DE, the model integrated with DE maintains higher accuracy throughout the entire training process, featuring a faster convergence speed and superior final performance. The experimental results demonstrate that the DE strategy can effectively enhance the model’s optimization capabilities and feature representation ability, indicating that the learnable parameter β can accelerate the convergence of the model.

#### 2.2.4. SR Module

The features after bi-temporal cross-attention are used for semantic segmentation prediction. However, cross-attention mainly focuses on the difference alignment between temporal phases, which may introduce noise while enhancing change information, affecting the accuracy of the semantic information of each temporal phase itself. The SR module performs local refinement on the semantic features of T1 and T2 to improve the quality of semantic segmentation. For the feature $F_{t}\;(t\;\in \;(T1,\;T2))$ of each temporal phase,

(18) $$R_{t}=\mathrm{ReLU}\;(\mathrm{BN}\;({\mathrm{Conv}}_{3\times 3}(\mathrm{ReLU}\;(\mathrm{BN}\;({\mathrm{Conv}}_{3\times 3}(F_{t}))))))$$

(19) $$F_{t}^{\mathrm{refined}}=F_{t}+\gamma_{t}\cdot R_{t}$$

where the scaling factor $\gamma_{t}$ is initialized to 0, and the parameters of the SR modules for the two temporal phases are independent of each other (without sharing weights), enabling them to learn semantic refinement strategies tailored to the characteristics of their respective temporal phases.

#### 2.2.5. Loss Functions

In this paper, multi-task loss functions are used as the loss functions for the corresponding tasks in the bi-temporal semantic segmentation task, change detection task and boundary detection task [27]. The formula of the bi-temporal semantic segmentation loss function *L_t_* (*t* = 1, 2) is as follows:

(20) $$L_{t}\;=-\frac{1}{N}\sum_{i=1}^{N}\sum_{C=1}^{C}y_{i}^{C}\;\log({\hat{y}}_{i}^{C})$$

where *N* indicates the number of image pixels; $y_{i}$ and ${\hat{y}}_{i}$ separately represent the categories of the ground truth and the actual predicted value, respectively, where i ∈ (0, 1, 2, … C), and *C* represents the number of semantic categories. Then, the loss function *L*_3_ for calculating the change detection region is

(21) $$L_{3}=-\frac{1}{N}\sum_{i=1}^{N}\left(W\;\times \;y_{c}\log\left({\hat{y}}_{c}\right)+(1-W)\;\times \;(1-{\hat{y}}_{c})\log\left(1-{\hat{y}}_{c}\right)\right)$$

where *W* indicates the weight of unchanged pixels (the proportion of negative samples to the total samples); $y_{c}$ and ${\hat{y}}_{c}$ are the change probabilities of the ground truth and the predicted region, respectively, with values ranging from 0 to 1. For the boundary detection task, the same loss function as in the change detection task is used ($L_{4}$ = $L_{3}$). In addition, to enhance the consistency between the change detection task and the semantic segmentation task, a semantic change loss function $L_{5}$ is used, which adopts the cosine function [28,30]:

(22) $$L_{5}=\left\{\begin{array}{c} 1\;-\cos\left(s_{1},s_{2}\right),\;y_{c}=\;0\\ \max(0,\;\cos\;(s_{1},s_{2})),\;y_{c}=\;1 \end{array}\right.$$

where *s*_1_ and *s*_2_ are the feature vectors of the semantic segmentation results of the previous and subsequent temporal phases, respectively. Finally, based on the uncertainty-weighted multi-task learning loss function of the baseline model, when the loss of a certain task is difficult to optimize, the uncertainty weight $\sigma_{i}^{2}$ will increase automatically to reduce the weight of this task. However, since the uncertainty weight $\sigma_{i}^{2}$ tends to infinity and $w_{\min}$ is initialized to 0, the weight $w_{i}=\frac{1}{2\sigma_{i}^{2}}$ will approach zero, which causes the corresponding task to be ignored. Therefore, by setting the minimum weight $w_{\min}$ = 0.2, the basic weight of each task is guaranteed, and the adaptive balance ability of the optimizer is maintained:

(23) $$L_{\mathrm{total}}=\sum_{i=1}^{5}(\max\;(\frac{1}{2\sigma_{i}^{2}},w_{\min})L_{i}\;+\;\log(1+\sigma_{i}^{2}))$$

## 3. Datasets and Accuracy Evaluation

### 3.1. Datasets

The method was tested on two public semantic change detection datasets, namely the SECOND and HRSCD datasets.

The SECOND dataset collects 4662 pairs of aerial images from multiple platforms and sensors, which are distributed in cities such as Hangzhou (30°17′ N, 120°10′ E), Chengdu (30°17′ N, 120°10′ E) and Shanghai (31°14′ N, 121°29′ E). Each image has a size of 512 × 512 pixels with a spatial resolution between 0.5 and 3 m and is annotated at the pixel level [33]. It includes 6 main land cover categories, namely ground, trees, low vegetation, water, buildings and playgrounds. For experimental convenience, we randomly select 3600 image pairs. These samples are randomly divided into training, validation and test sets at a ratio of 4:1:1, and samples with a proportion of zero change pixels are discarded. Finally, 2375, 593 and 593 image pairs are obtained for training, validation and testing, respectively.

The HRSCD dataset contains 291 pairs of images with a spatial size of 10,000 pixels × 10,000 pixels [34]. All image pairs have a spatial resolution of 0.5 m and were collected in multiple cities in France in 2006 and 2012. The dataset includes five types of semantic change, namely wetlands, agricultural areas, forests, water and artificial surfaces. Considering the extremely large image size and severe class imbalance in the dataset (where unchanged pixels account for over 99.2% of all pixels), for experimental convenience, we select images from the D35 folder, containing a total of 190 image pairs. For data preprocessing, all original images and corresponding ground truths are directly cropped into non-overlapping 512 × 512 pixel patches without overlap or stride by uniformly dividing the original images along the width and height directions. Patches with zero change pixels in the label maps are discarded. To ensure full reproducibility, a fixed random seed of 1 is used throughout all experiments for data splitting and model initialization. The processed samples are randomly split into training, validation and test sets with a ratio of 8:1:1. In the inference phase, random flipping augmentation is adopted. All experiments are conducted on a single NVIDIA A100 GPU. The average training time for each model is about 12 h under the same experimental configuration. Ultimately, we obtain 4115, 152 and 378 image pairs for training, validation and testing, respectively.

### 3.2. Accuracy Evaluation

For the change detection task, the prediction results of the model can be divided into four basic scenarios; let true positive (TP), false positive (FP), true negative (TN) and false negative (FN) represent the numbers of correctly identified changes, falsely identified changes, correctly detected unchanged pixels and missed changes, respectively. To comprehensively evaluate the model’s performance, this study adopts a corresponding set of evaluation metrics for different types of semantic change detection tasks.

Pre (Precision): It measures the proportion of pixels predicted as changed that are actually changed. The formula is as follows:

(24) $$\mathrm{Pre}\;=\;\frac{\mathrm{TP}}{\mathrm{TP}\;+\;\mathrm{FP}}$$

Rec (Recall): It measures the proportion of actually changed pixels that are correctly detected among all truly changed pixels. The formula is as follows:

(25) $$\mathrm{Rec}\;=\;\frac{\mathrm{TP}}{\mathrm{TP}\;+\;\mathrm{FN}}$$

F1 (F1-Score): It is the harmonic mean of precision and recall, which comprehensively reflects model performance by integrating the two indicators. The formula is as follows:

(26) $$F1\;=\;\frac{2\;\times \;\left(\mathrm{Pre}\;\times \;\mathrm{Rec}\right)}{\left(\mathrm{Pre}\;+\;\mathrm{Rec}\right)}$$

mIoU (Mean Intersection over Union): IoU measures the overlap degree between the predicted change region and the true change region. We calculate the average of the non-change IoU (IoU_nc_) and the change IoU (IoU_c_) to obtain the mIoU value:

(27) $${\mathrm{IoU}}_{c}\;=\;\frac{\mathrm{TP}}{\mathrm{TP}\;+\;\mathrm{FP}\;+\;\mathrm{FN}}$$

(28) $${\mathrm{IoU}}_{nc}=\frac{\mathrm{TN}}{\mathrm{TP}+\mathrm{FP}+\mathrm{FN}}$$

(29) $$\mathrm{mIoU}=\frac{{\mathrm{IoU}}_{c}+{\mathrm{IoU}}_{\mathrm{nc}}}{2}$$

OA (Overall Accuracy): It evaluates the overall proportion of all pixels (changed and unchanged) that are correctly classified:

(30) $$\mathrm{OA}\;=\;\frac{\mathrm{TP}+\mathrm{TN}}{\mathrm{TP}+\mathrm{FP}+\mathrm{TN}+\mathrm{FN}}$$

SeK (Separated Kappa): It is used to measure the consistency between classification results and ground truth labels. Let *S_ij_* be the count of pixels belonging to label *i* identified as category *j*, where i,j∈{0,1,…,G−1} (*G* denotes the number of classified categories). Let *S =* {*S_ij_*} be the confusion matrix of the identified results and ground truth, followed by the SeK value:

(31) $$\mathrm{SeK}\;=\;e^{{\mathrm{IoU}}_{c}1}\cdot \frac{\rho \;-\hat{\rho }}{1\;-\hat{\rho }}$$

(32) $$\rho =\frac{\sum_{i=1}^{G}S_{\mathrm{ii}}}{\sum_{i=0}^{G}\sum_{j=0}^{G}S_{\mathrm{ij}}-S_{00}}$$

(33) $$\hat{\rho }=\frac{\sum_{j=0}^{G}\left({\hat{S}}_{j+}\cdot {\hat{S}}_{+j}\right)}{{(\sum_{i=0}^{G}\sum_{j=0}^{G}S_{\mathrm{ij}}-S_{00})}^{2}}$$

where ρ and $\hat{\rho }$ represent the observed agreement rate and expected agreement rate, respectively. *S_+j_* and *S_j+_* denote the column sum and row sum that exclude the unchanged pixels *S*_00_ from the matrix *S*.

## 4. Experiments

### 4.1. Experimental Environment Configuration

All experiments in this paper are trained and run on a server. The server operating system is Linux, equipped with two Intel(R) Xeon(R) Gold 6326 CPUs @ 2.90 GHz and an NVIDIA A100 GPU. The PyTorch 2.0.0 deep learning framework is used, configured with CUDA 12.4 for model training. The optimizer for all experiments is set to AdamW, the learning rate is set to 1 × 10^−4^, the number of training epochs is set to 100 for both datasets, and the batch size is set to 6.

### 4.2. Performance Comparison

To evaluate the semantic change detection performance of the SRDFNet proposed in this paper, comparative experiments were conducted on the SECOND dataset between this model and three other semantic change detection models, namely BGSNet, BiSRNet [28] and HGINet [30]. The accuracy metrics of the four models on the test dataset were calculated, as shown in Table 1. It can be seen from Table 1 that SRDFNet achieves the highest overall accuracy and recall rates among the four models. SRDFNet obtains the highest OA value of 87.64%, indicating its optimal performance in global pixel classification accuracy. SRDFNet also achieves the best result for the recall metric, reaching 65.27%, which demonstrates that the model can detect more real change regions with the lowest missed detection rate. Other metrics, namely the mIoU, SeK and F1, are improved by 0.73%, 1.44% and 0.81%, respectively, compared with the baseline model BGSNet. However, SRDFNet achieves mIoU and F1 scores of only 70.31% and 60.25%, which are lower than those of BiSRNet (71.46%, 60.46%) and HGINet (71.25%, 60.90%). In terms of the SeK metric, SRDFNet reaches 20.36%, slightly outperforming BiSRNet (20.12%) but inferior to HGINet (20.68%). These results reveal several limitations of SRDFNet: (1) insufficient consistency in global semantic segmentation; (2) limited capabilities to mine features of small samples and weak change regions; (3) the overall balanced performance for change detection requires further optimization.

**Figure 5:**
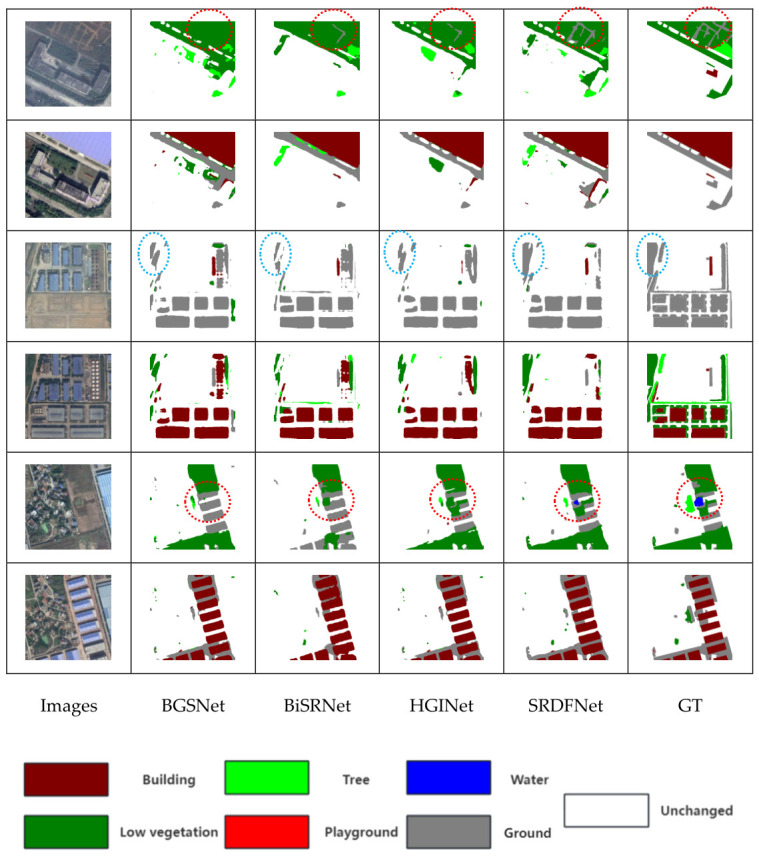
Semantic change detection result maps of various methods on the SECOND dataset.

Figure 5 illustrates the detection results of different methods on the SECOND dataset. The proposed SRDFNet accurately identifies the changed categories and regions, demonstrating the model’s capabilities in perceiving local semantic details; as shown by the blue boxes in Figure 5, SRDFNet achieves a lower omission rate. Especially in terms of geometric features, as indicated by the red boxes in Figure 5, our model produces regular boundaries and clear contours. Moreover, it exhibits superior anti-interference performance in complex scenes with mixed semantic categories including ground, vegetation and water. The model shows greater advantages over other methods in extracting boundaries and shapes. Nevertheless, SRDFNet still has certain limitations: it is prone to false detection when identifying small-scale objects, especially for shadow regions surrounding buildings.

To verify the generalization ability of the proposed semantic change detection model SRDFNet, this model was also compared with the other three methods on the HRSCD dataset. As can be seen from Table 2, our method achieves the best performance in all five metrics: OA, mIoU, SeK, F1, and recall. Especially in the SeK metric, it outperforms BGSNet, BiSRNet and HGINet by 9.69%, 41.81% and 29.65%, respectively.

**Figure 6:**
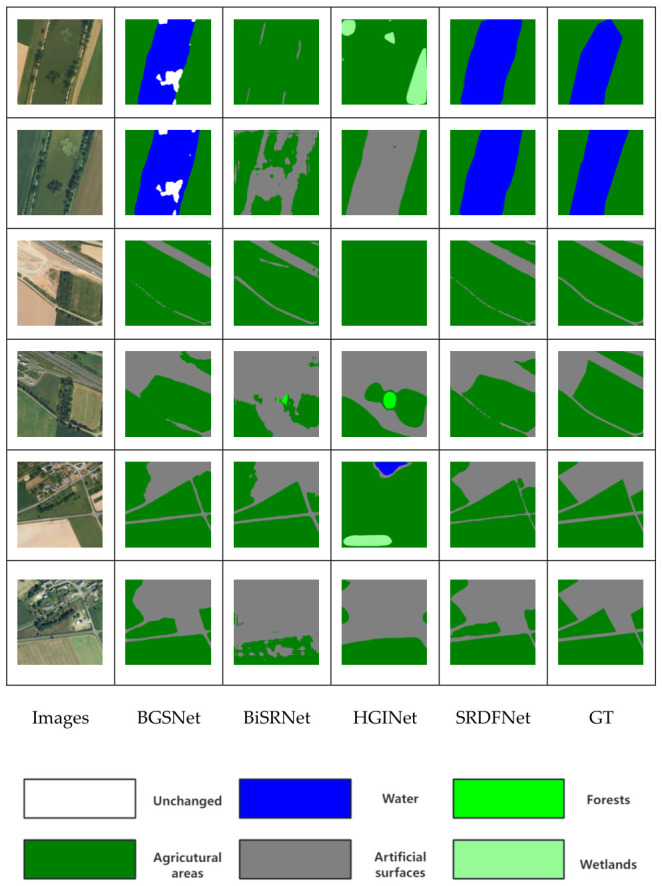
Semantic change detection results of various methods on the HRSCD dataset.

We also present the comparative SCD results for the four models on the HRSCD dataset, as shown in Figure 6. Our method effectively identifies various “from–to” change information. Meanwhile, compared with other models, our model significantly reduces missed detections and false detections, achieving the best accuracy performance and the highest semantic segmentation precision. The results intuitively demonstrate that the semantic segmentation masks obtained by SRDFNet are more accurate, with fewer omissions and clearer boundaries. From the quantitative results, a noticeable performance gap can be observed between the proposed SRDFNet and competitive methods including BiSRNet and HGINet. To guarantee a fair comparison, all baseline methods were evaluated under completely unified experimental settings: all experiments adopted the AdamW optimizer with a fixed learning rate of 1 × 10^−4^. Specifically, we strictly followed the official public implementations of all compared methods, kept the same backbone network and consistent pre-trained weight file (pvt_v2_b2.pth [31]) across all models and avoided the additional re-tuning of the hyperparameters for individual methods to eliminate experimental bias. In addition to the qualitative visualization results, we find that the obvious performance gap on the HRSCD dataset is mainly caused by the annotation noise and severe class imbalance inherent in the dataset. On the one hand, partial low-quality and inaccurate annotations introduce inevitable interference in model training. On the other hand, severe semantic confusion exists between visually and spectrally similar categories (e.g., forests and wetlands) in HRSCD; the analogous spectral distribution and visual characteristics further enlarge the performance discrepancy among the different methods.

### 4.3. Ablation Studies

To evaluate the effectiveness of the three constructed modules (HGM, DE and SR) in SRDFNet, we selected the SECOND dataset for ablation experiments. To ensure reliability, all experiments were conducted under consistent settings, including the same learning rate and pre-trained weight file (pvt_v2_b2.pth [31]). The final results are shown in Table 3. The combination of HGM + DE + SR achieves the best performance in OA, SeK and F1, and it is only slightly poorer than the DE + SR combination in the mIoU. Meanwhile, in the ablation experiment with the HGM module alone, the OA reaches 84.13%, which is slightly lower than the baseline of 84.18%. This indicates that the HGM alone cannot improve the OA metric. Instead, the three designed modules interact and mutually promote each other to boost the final performance. Overall, the ablation results sufficiently validate the effectiveness of each proposed module.

**Figure 7:**
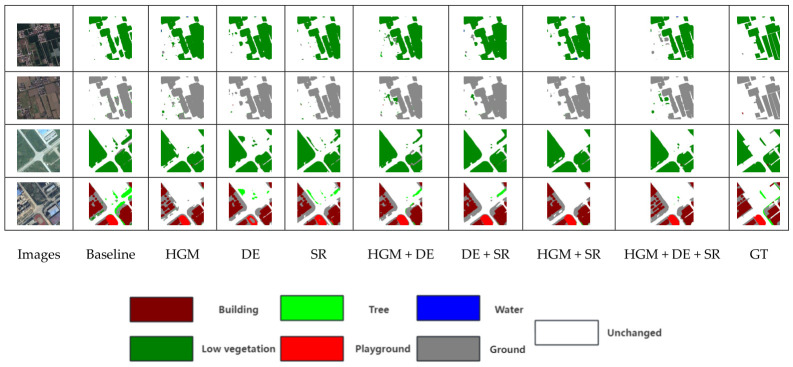
Comparison of semantic change results of HGM, DE and SR combined modules on the SECOND dataset.

Figure 7 presents the semantic change detection results for different combinations of the three modules (HGM, DE and SR) on the SECOND dataset. In simple change scenarios dominated by low vegetation and ground, a single module can already yield significant performance improvements, while the benefits of multi-module combinations are mainly reflected in noise suppression. In contrast, in scenarios containing complex semantic categories such as buildings and playgrounds, the limitations of individual modules (i.e., blurred boundaries and category confusion) become apparent. The multi-module combination, especially the full model with HGM + DE + SR, achieves high-precision semantic change recognition through the complementary advantages of each module, demonstrating the robustness of the proposed modules in complex urban scenarios.

Similarly, we also selected the HRSCD dataset for ablation experiments. To ensure reliability, all experiments were conducted under consistent settings. The final results are shown in Table 4. The combination of HGM + DE + SR achieves the best performance in OA, SeK and F1 and is only slightly poorer than the HGM + SR combination in the mIoU. Meanwhile, other combinations also show certain improvements over the baseline, which verifies the effectiveness of the three modules.

**Figure 8:**
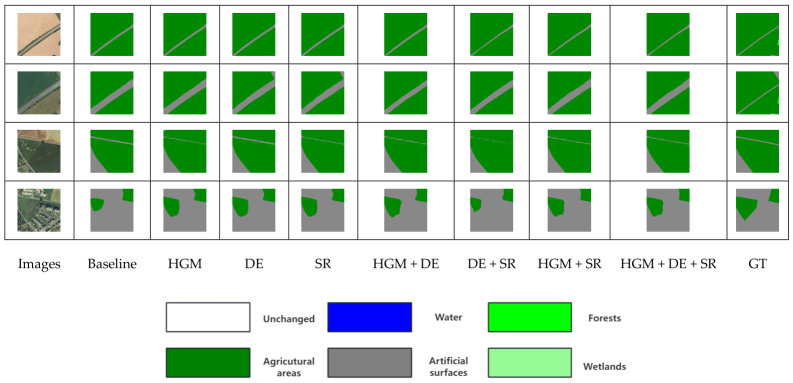
Comparison of semantic change results of HGM, DE and SR combined modules on the HRSCD dataset.

Figure 8 presents the semantic change detection results for different combinations of the three modules (HGM, DE and SR) on the HRSCD dataset. Compared with the baseline model, the introduction of any single module (HGM/DE/SR) improves the model’s recognition accuracy for the boundaries of linear features (i.e., roads), the contours of areal features and semantic categories in high-resolution scenarios. Each module plays a distinct role with its own focus: the HGM achieves remarkable effects in noise suppression and continuity optimization for long-distance linear features; the DE module significantly enhances the feature discriminability between different semantic categories; and the SR module excels in high-resolution detail recovery and contour accuracy optimization.

### 4.4. Comprehensive Efficiency Analysis of the Models

We conducted complexity experiments on different algorithm models and compared the proposed SRDFNet with mainstream models from four dimensions: the number of parameters (Params), floating-point operations (FLOPs), inference time (Inference) and FPS. The unit of FLOPs is the memory access cost (Mac), and FPS represents the number of images that can be processed per second. As shown in Table 5, SRDF has the lowest FLOPs, which is 61.0% lower than that of BGSNet, showing significant lightweight advantages, but the inference time and FPS have not been improved. It is worth noting that BiSRNet leads in the three indicators of Params, Inference and FPS, but its FLOPs reaches the highest of 190.298 G. Overall, BiSRNet has optimal real-time performance but serious computational redundancy, making it suitable for high-computing-power and real-time scenarios; although SRDFNet has low inference efficiency, it has significant lightweight potential and is suitable for deployment on low-computing-power devices.

We further conduct a complexity analysis of the three proposed modules, HGM, DE and SR, as shown in Table 6. The ablation results in the table illustrate the effectiveness and computational efficiency of the proposed HGM, DE and SR modules. Compared with the baseline, all variant models only introduce a slight increase in parameters and FLOPs, while the GPU memory consumption remains almost unchanged. The inference latency of all models is maintained at the same millisecond level, demonstrating that the embedded modules bring negligible computational overhead. In terms of quantitative accuracy, each individual module achieves a consistent F1 improvement over the baseline. Moreover, the combined strategy of multiple modules yields further performance gains, and the integrated SRDF_HGM+DE+SR_ obtains the highest F1 score of 60.25, validating the positive complementary effect and rationality of the three designed modules.

## 5. Conclusions

This paper addresses two long-standing issues in semantic change detection: (1) insufficient multi-task collaboration—existing methods typically treat the semantic segmentation branch and the change detection branch as mutually independent subtasks, neglecting their intrinsic coupling in the feature space and thus failing to enable positive information complementarity among subtasks; (2) a lack of semantic consistency—during cross-level feature fusion and bi-temporal interaction, it is difficult to preserve the topological consistency of identical land-cover categories across space and the semantic integrity within each individual phase, leading to inter-class confusion and blurred boundaries.

To address the above issues, this paper proposes SRDFNet. In order to fill the research gap regarding insufficient semantic interaction modeling and poor discrimination of difference features in existing methods, SRDFNet implements a collaborative optimization framework composed of three core modules, HGM, DE and SR, which function in feature encoding, difference modeling and semantic decoding, respectively. This comprehensive design enables the model to achieve the joint optimization of multiple key subtasks during training, yielding substantial performance improvements that transcend conventional structural refinements of SCD models.

Extensive experiments were conducted on the SECOND and HRSCD datasets, and the conclusions are as follows:

(1) The three constructed modules (HGM, DE and SR) significantly improve the performance of the baseline model. Compared with the BGSNet, our SRDFNet achieves consistent performance gains on both the SECOND and HRSCD datasets. Specifically, the OA is improved by 1.34% and 3.96%, the SeK by 1.44% and 9.69% and the F1 by 0.81% and 1.33% on the two datasets, respectively.
(2) The model demonstrates adaptability to multi-task loss optimization. On both the SECOND and HRSCD datasets, SRDFNet exhibits strong generalization abilities; it consistently outperforms all comparative models and achieves the best overall performance across all evaluation metrics on the HRSCD dataset.

Looking ahead, the fusion of multi-source data has gradually become a mainstream research direction. Meanwhile, multi-source data fusion can also provide richer feature representations. We plan to apply our model to change detection using multi-source remote sensing data—for example, fusing optical images with synthetic aperture radar (SAR) images. We will design cross-modal feature alignment and fusion modules to perform semantic change detection in a unified feature space and explore cross-domain generalization.

## Figures and Tables

**Table 1:** Performance comparison of different SCD methods on the SECOND dataset (the best metric values are marked in bold).

Method	OA (%)	mIoU (%)	SeK (%)	F1 (%)	Recall (%)
BGSNet	85.30	69.58	18.92	59.44	62.55
BiSRNet [28]	87.35	**71.46**	20.12	60.46	56.73
HGINet [30]	87.11	71.25	**20.68**	**60.90**	58.56
SRDFNet	**87.64**	70.31	20.36	60.25	**65.27**

**Table 2:** Performance comparison of different SCD methods on the HRSCD dataset (the best metric values are marked in bold).

Method	OA (%)	mIoU (%)	SeK (%)	F1 (%)	Recall (%)
BGSNet	94.17	48.74	64.08	86.53	84.18
BiSRNet [28]	60.75	49.73	31.96	60.85	60.98
HGINet [30]	72.70	49.80	44.12	72.85	73.00
SRDFNet	**98.1** **3**	**52.67**	**73.77**	**88.86**	**88.18**

**Table 3:** Ablation results of the three modules HGM, DE and SR on the SECOND dataset (the best metric values are marked in bold).

HGM	DE	SR	OA (%)	mIoU (%)	SeK (%)	F1 (%)
			84.18	68.85	18.69	58.55
√			84.13	69.38	19.74	58.99
	√		85.12	70.21	20.16	59.89
		√	84.57	69.73	19.83	59.41
√	√		84.80	69.99	20.18	59.82
	√	√	85.08	**70.36**	20.23	59.90
√		√	85.22	70.08	19.68	59.57
√	√	√	**85.29**	70.31	**20.36**	**60.25**

**Table 4:** Ablation results of the three modules HGM, DE and SR on the HRSCD dataset (the best metric values are marked in bold).

HGM	DE	SR	OA (%)	mIoU (%)	SeK (%)	F1 (%)
			84.04	48.74	64.08	86.64
√			87.20	52.37	71.39	88.32
	√		85.57	49.22	67.82	87.66
		√	87.26	49.57	73.37	87.78
√	√		87.93	52.31	73.84	88.80
	√	√	86.36	51.58	69.95	87.51
√		√	87.23	**53.03**	71.96	88.06
√	√	√	**88.34**	52.66	**74.99**	**89.18**

**Table 5:** Comparative experiments on multiple efficiency indicators of the models (the best metric values are marked in bold).

Model	Params (M)	FLOPs (G)	Inference (ms)	FPS (Picture/s)
BGSNet	25.185	117.049	35.94 ± 0.73	27.83
BiSRNet [28]	**23.376**	190.298	**11.43 ± 2.37**	**87.48**
HGINet [30]	27.703	50.637	29.39 ± 4.39	34.03
SRDFNet	25.070	**45.664**	33.92 ± 0.43	26.96

**Table 6:** Comparison of model complexity and quantitative performance with different module combinations (the best metric values are marked in bold).

Model	Params (M)	FLOPs (G)	Inference (ms)	Mem (MB)	F1
Baseline	**24.964**	**44.297**	**30.71 ± 0.46**	**4225.1**	59.17
SRDF_HGM_	24.991	44.355	33.83 ± 0.48	4225.2	59.73
SRDF_DE_	25.007	44.993	30.63 ± 0.42	4225.3	59.77
SRDF_SR_	25.001	44.909	31.16 ± 0.41	4225.3	59.40
SRDF_HGM+DE_	25.033	45.051	33.18 ± 0.41	4225.4	59.82
SRDF_DE+SR_	25.044	45.606	30.75 ± 0.44	4225.5	60.06
SRDF_HGM+SR_	25.028	44.967	34.21 ± 0.39	4225.4	59.58
SRDF_HGM+DE+SR_	25.070	45.664	33.92 ± 0.43	4225.6	**60.25**

## Data Availability

The data presented in this study are available upon request from the corresponding author.

## Abbreviations

The following abbreviations are used in this manuscript:

BGSNet	Boundary-Guided Siamese Network
SRDFNet	Semantic Refinement and Differential Features Network
HGM	Hierarchical Graph Module
DE	Difference Enhancement
SR	Semantic Refine
MLP	Multilayer Perceptron
SCD	Semantic Change Detection
BCG	Boundary-Contextual Guidance
IDET	Iterative Difference Enhancement
